# Initial Identification of UDP-Glucose Dehydrogenase as a Prognostic Marker in Breast Cancer Patients, Which Facilitates Epirubicin Resistance and Regulates Hyaluronan Synthesis in MDA-MB-231 Cells

**DOI:** 10.3390/biom11020246

**Published:** 2021-02-09

**Authors:** Daiana L. Vitale, Ilaria Caon, Arianna Parnigoni, Ina Sevic, Fiorella M. Spinelli, Antonella Icardi, Alberto Passi, Davide Vigetti, Laura Alaniz

**Affiliations:** 1 Laboratorio de Microambiente Tumoral, Centro de Investigaciones Básicas y Aplicadas (CIBA), Universidad Nacional del Noroeste de la Provincia de Buenos Aires, Junín 6000, Argentina; dlvitale@comunidad.unnoba.edu.ar (D.L.V.); isevic@comunidad.unnoba.edu.ar (I.S.); fspinelli@comunidad.unnoba.edu.ar (F.M.S.); antoicardi@gmail.com (A.I.); 2 Centro de Investigaciones y Transferencia del Noroeste de la Provincia de Buenos Aires (CITNOBA), UNNOBA-UNSAdA-CONICET, Junín 6000, Argentina; 3 Dipartimento di Medicina e Chirurgia, Università degli Studio dell’Insubria, 21100 Varese, Italy; i.caon@uninsubria.it (I.C.); a.parnigoni@uninsubria.it (A.P.); alberto.passi@uninsubria.it (A.P.)

**Keywords:** UDP-glucose dehydrogenase, hyaluronan, epirubicin, drug resistance, extracellular matrix, breast cancer

## Abstract

UDP-glucose-dehydrogenase (UGDH) synthesizes UDP-glucuronic acid. It is involved in epirubicin detoxification and hyaluronan synthesis. This work aimed to evaluate the effect of UGDH knockdown on epirubicin response and hyaluronan metabolism in MDA-MB-231 breast cancer cells. Additionally, the aim was to determine UGDH as a possible prognosis marker in breast cancer. We studied UGDH expression in tumors and adjacent tissue from breast cancer patients. The prognostic value of UGDH was studied using a public Kaplan–Meier plotter. MDA-MB-231 cells were knocked-down for UGDH and treated with epirubicin. Epirubicin-accumulation and apoptosis were analyzed by flow cytometry. Hyaluronan-coated matrix and metabolism were determined. Autophagic-LC3-II was studied by Western blot and confocal microscopy. Epirubicin accumulation increased and apoptosis decreased during UGDH knockdown. Hyaluronan-coated matrix increased and a positive modulation of autophagy was detected. Higher levels of UGDH were correlated with worse prognosis in triple-negative breast cancer patients that received chemotherapy. High expression of UGDH was found in tumoral tissue from HER2^-^-patients. However, UGDH knockdown contributes to epirubicin resistance, which might be associated with increases in the expression, deposition and catabolism of hyaluronan. The results obtained allowed us to propose UGDH as a new prognostic marker in breast cancer, positively associated with development of epirubicin resistance and modulation of extracellular matrix.

## 1. Introduction

An increased understanding of mechanisms that favor the aggressive behavior of tumor cells and the role of the tumor microenvironment provide insights into novel treatment strategies for breast cancer. The extracellular matrix (ECM) is one of the most important components of the tumor microenvironment that can directly modulate cell growth, survival, migration, immune response and drug resistance [1,2]. Among the main molecular components of ECM are glycosaminoglycans (GAGs) and proteoglycans (PGs), which have been shown to play fundamental roles in different physiological processes and malignancies [3,4,5].

UDP-glucuronic acid (UDP-GlcUA) is a precursor of several GAGs and PGs present in the ECM. UDP-GlcUA is formed by the oxidation of UDP-glucose through the catalytic action of the UDP-glucose dehydrogenase (UGDH) enzyme [6,7,8]. This reaction is part of glucose metabolism, since glucose is converted to glucose-1-phosphate and then to UDP-glucose, an active form of glucose, which is further transformed to UDP-GlcUA. Once UDP-GlcUA is formed, it can be a substrate of different divergent pathways [9]. It is involved in hyaluronan (HA) synthesis, through the catalytic action of HA synthases (HAS1, HAS2 and HAS3) which binds UDP-GlcUA to N-acetyl-glucosamine. Besides, UDP-GlcUA is a precursor for the polymerization of heparan sulfate chains [10]. Due to the conversion to UDP-xylose, UDP-GlcUA initiates the production of different proteoglycans, such as chondroitin sulfate [11]. On the other hand, the UGDH enzyme and its product UDP-GlcUA have important roles in drug detoxification and clearance, and represent a protective mechanism for improved elimination of lipophilic xenobiotics from the organism [12,13].

Specifically, UDP-GlcUA plays a key role during the metabolism and elimination of chemotherapeutic drugs used in the treatment of hormone-resistant breast cancer, such as the anthracycline epirubicin (EPI) [14,15]. EPI is extensively metabolized in the liver and the main detoxifying pathway occurs through the formation of a glucuronide form of EPI (4’-*O*-b-d-glucuronyl-4′-epi-doxorubicin) via a glucuronidation reaction. Glucuronidation is carried out by UDP-glucuronosyl-transferases enzymes (UGTs), classified into subfamilies based on their amino acid sequence homology [16]. EPI is mainly glucuronidated by the addition of one molecule of UDP-glucuronic acid (UDP-GlcUA), through the action of the specific UGT called UGT2B7 [17].

Drug resistance limits the efficacy of anthracyclines and other antineoplastic therapies [18]. In particular, the development of resistance to EPI can occur via different mechanisms, including P-glycoprotein-mediated resistance, changes in topoisomerase II activity, induction of heat shock proteins and inhibition of apoptotic pathways [19]. Since EPI is glucuronidated by UGT2B7, alteration of the availability of its precursor or substrate could have potential impacts on EPI systemic clearance and efficacy. Indeed, it has been demonstrated that EPI upregulates UGT2B7 expression in hepatocellular carcinoma HepG2 and Huh7 cells via p53 [20], suggesting that detoxifying genes are activated by the p53-mediated pathway to clear genotoxic agents. It has also been observed that cell autophagy protects MCF-7 breast cancer cells from EPI-induced apoptosis and facilitates EPI resistance development, acting as a pro-survival factor [21].

In cancer development it is well known that HA expression is usually altered, affecting several mechanisms associated with cell proliferation and survival, invasion, angiogenesis and multidrug resistance, among others. Even more, it has been demonstrated that HA also affects immune cells’ recruitment and inflammation in the tumor context [22,23,24,25,26]. As mentioned above, HA metabolism is in part dependent on UGDH expression and activity, since it controls the availability of HA precursor UDP-GlcUA. This enzyme has been proposed as a novel candidate biomarker of prostate cancer that may complement the development of a multi-biomarker panel for detecting tumor transformation within the adjacent tumor tissue [27]. Even more, it has been determined that the treatment of colorectal carcinoma HCT-8 cells with either UGDH-specific small interference RNA (siRNA) or HA synthesis inhibitor 4-methylumbelliferone (4-MU) effectively delays cell aggregation [2], and the authors proposed UGDH as a potential target for therapeutic intervention of colorectal cancers. However, the importance of the glucuronidation reaction and the role of the UGDH enzyme in breast cancer treatment have not yet been studied.

Furthermore, possible modulations of these mechanisms by EPI in the tumor microenvironment could be clinically relevant. Therefore, this work aimed to evaluate the role of UGDH during EPI treatment in a triple-negative breast cancer model MDA-MB-231. We evaluated the effect of silencing the UGDH gene with a specific siRNA on the EPI response using that aggressive breast cancer cell line, by studying cellular processes ranging from cell survival to modulation of extracellular matrix composition. Besides, we studied the expression of UGDH in breast cancer patients, both in tumors and adjacent normal tissue, and the association of its expression with patient’s survival to propose it as a future prognostic marker.

## 2. Materials and Methods

### 2.1. Reagents

Amaxa^®^ Cell Line Nucleofector^®^ Kit V was purchased from Lonza (Cologne, Germany). High glucose Dulbecco’s modified eagle’s medium (DMEM) was from EuroClone S.p.A. (Milan, Italy). EPI was purchased from Selleckchem (Houston, TX, USA). Anti-β-catenin antibody was purchased from Merck KGaA, (Darmstadt, Germany). A specific antibody against glyceraldehyde-3-phosphate dehydrogenase (GAPDH) was from NeoBioLab (Boston, MA, USA). Anti-phosphorylated Akt (Ser473, Ser472 and Ser474) antibody was purchased from R&D System (Minneapolis, MN, USA) and anti-rabbit secondary horseradish peroxidase (HRP) antibody was purchased from Santa Cruz Biotechnology (Dallas, TX, USA). Annexin V-FITC apoptosis detection kit was from BioVision (San Jose, CA, USA). LDH-cytotoxicity Assay Kit was purchased from Abcam (Cambridge, MA, USA).

### 2.2. Cell Culture

To carry out the in vitro experiments, it was decided to use one of the most used triple negative breast cancer models characterized as highly aggressive, invasive and poorly differentiated in phenotype [28]. This cell line also represents a suitable model to study tumors with limited treatment options. The immortalized human breast adenocarcinoma cell line MDA-MB-231 (ATCC^®^ HTB-26) was maintained in exponential growth by serial passages in DMEM-high glucose medium supplemented with 2 mM L-glutamine, 100 IU penicillin, 100 µg/mL streptomycin and 10% *v*/*v* of fetal bovine serum (FBS) in a humidified incubator at 37 °C and 5% CO_2_. During all cell cultures, periodic checkups of cell morphology and growth rate were performed, and the strict control of cell line passages (5–10th passage). The MDA-MB-231 cell line was authenticated by Northgene Ltd. Company (Newcastle, UK), using highly sensitive DNA testing for short tandem repeats (STR). The cell line was also analyzed to discard the presence of mycoplasma contamination by PCR [29].

### 2.3. Transfection and EPI Treatment

MDA-MB-231 cells were plated in a 6 well-plate (1 × 10^6^) and transfected through nucleoporation with 30 nM of one sequence of UGDH small interference RNA (siRNA, siUGDH, ThermoFisher, Monza, Italy) or a negative control siRNA (siSCR ThermoFisher) having a random sequence, using the Amaxa^®^ Cell Line Nucleofector^®^ Kit V. After 24 h of incubation, 1 µM of EPI (EPI) was added to complete 48 h of incubation after transfection in combination with both siRNAs (siUGDH + EPI and siSCR + EPI). EPI treatment was performed to compare the results caused by the drug, and a control without transfection (basal). During both treatments, cells were maintained in a humidified incubator at 37 °C and 5% CO_2_. Subsequently, supernatants were collected and conserved at –80 °C until their use.

### 2.4. Viability Assay

In order to evaluate possible alterations in cell viability, after the transfection with siRNA against UGDH and EPI treatment, MDA-MB-231 cells (1 × 10^3^) were plated in a 96-well plate and incubated at 37 °C and 5% CO_2_ for 24 h. All samples were treated with 50 µL per well of 3-(4,5-Dimethyl-2-thiazolyl)-2,5-diphenyl-2H-tetrazolium·bromide (Serva) and incubated at 37 °C for 4 h. After adding 200 µL/well of DMSO to dissolve crystals, optical density (OD) was quantified by spectrophotometer (Bio-Rad Laboratories, Hercules, CA, USA) at 570 nm.

### 2.5. Cytotoxicity Assay

After transfection and EPI treatment, cellular cytotoxicity was evaluated through the measurement of the activity of lactate dehydrogenase (LDH) enzyme released from damaged cells using the specific LDH Cytotoxicity Assay Kit (Abcam, Cambridge, MA, USA). LDH enzyme oxidizes lactate to pyruvate, which reacts with a tetrazolium salt (INT) to form formazan. Supernatants of transfected and treated MDA-MB-231 cells were analyzed following the manufacturer’s protocol.

### 2.6. Epirubicin Accumulation Assay

EPI is a single molecule capable of emitting fluorescence detectable by flow cytometry (550–600 nm). This property allows the determination of EPI intracellular accumulation as we previously described [29]. MDA-MB-231 cells (5 × 10^5^) were transfected and treated as mentioned above and EPI fluorescence was collected through a 564–606 nm band-pass filter. Samples were analyzed using a FACS Aria II cytometer and data was evaluated using FlowJo 5 software (Becton, Dickinson and Company, Franklin Lakes, NJ, USA).

### 2.7. Apoptosis Detection Assay

To evaluate apoptosis, MDA-MB-231 cells (5 × 10^5^) were transfected and treated as mentioned above. After culture procedures, cells were stained with annexin V-FITC reagent for 30 min at room temperature (RT) following the manufacturer’s protocol. Samples were analyzed using FACS Aria II cytometer and data were evaluated using FlowJo 5 software (Becton, Dickinson and Company, Franklin Lakes, NJ, USA).

### 2.8. RT-qPCR

Total RNA from MDA-MB-231 cells (1 × 10^6^) was extracted using PureLink^®^ RNA Mini Kit Life Technologies (Life Technologies, ThermoFisher, Monza, Italy). RNA integrity and quantification were assessed by a spectrophotometry system, measuring OD260 and OD280 in Nanodrop^®^ instruments. Two µg of RNA were reverse transcribed using High Capacity cDNA Reverse Transcription Kit (Applied Biosystems, ThermoFisher, Monza, Italy).

cDNAs were analyzed by quantitative real-time PCR (RT-qPCR) using FastStart SYBR Green: UGT2B7 (Fw: 5′ GGA GAA TTT CAT CAT GCA ACA GA 3′ and Rv: 5′ CAG AAC TTT CTA GTT ATG TCA CCA AAT ATT G 3′); ABCC1 (Fw: 5′ AAG TCG GGG CAT ATT CCT G 3′ and Rv: 5′ TGA AGA CTG AAC TCC CTT CCT C 3′); ABCC2 (Fw: 5′ AAA TCC AGG ACC AAG AGA TCC 3′ and Rv: 5′ TGT GGC TTG TCC AGA GTC TTC 3′); ABCG2 (Fw: 5′ GCT GCA AGG AAA GAT CCA AG 3′ and Rv: 5′ CAG AGT GCC CAT CAC AAC ATC 3′); VEGF (Fw: 5′ CTA CCT CCA CCA TGC CAA GT 3′ and Rv: 5′ GCA GTA GCT GCG CTG ATA GA 3′); EGF (Fw: 5′ TGA TAA GCG GCT GTT TTG G 3′ and Rv: 5′ CAC CAA AAA GGG ACA TTG C 3′); HYAL1 (Fw: 5′ GGC TAT GAG GAA ACT GAG TCA C 3′ and Rv: 5′ TAG GAG TGC AAG GGC TGT AC 3′); HYAL2 (Fw: 5′ ATC TCT ACC ATT GGC GAG AGT G 3′ and Rv: 5′ ATC TTT GAG GTA CTG GCA GGT C 3′); HYAL3 (Fw: 5′ TAT GTC CGC CTC ACA CAC C 3′ and Rv: 5′ CTG CAC TCA CAC CAA TGG AC 3′) and LC3-II (Fw: 5′ AGC AGC ATC CAA CCA AAA TC 3′ and Rv: 5′ CTG TGT CCG TTC ACC AAC AG 3′) or Taqman^®^ probes: UGDH (Hs00163365_m1), HAS2, (Hs00193435_m1) and HAS3 (Hs00193436_m1) assays (Applied Biosystems).

PCR conditions for SYBR Green reactions were 90 s at 94 °C and then 40 cycles of 30 s at 94 °C, 30 s at 60 °C and 30 s at 72 °C. PCR conditions for probes reactions were 2 min at 50 °C, 10 min at 95 °C and then 40 cycles of 15 s at 95 °C and 60 s at 60 °C. All the assays were performed using Abi 7000 Sequence Detection System instrument (Applied Biosystems, ThermoFisher, Monza, Italy). Results were normalized using β-actin (Fw: 5′ GGG GCT GCC CAG AAC ATC AT 3′ and Rv: 5′ GCC TGC TTC ACC ACC TTC TTG 3′) as a reference gene and all determinations were performed as duplicates in three separated experiments. A non-template control (NTC) was correspondingly added during every assay.

### 2.9. Protein Extracts and Western Blot

To analyze protein expression, MDA-MB-231 cells (1 × 10^6^) were transfected and treated with EPI as described above and then were lysed with RIPA (Radioimmunoprecipitation assay) lysis buffer (150 mM NaCl, 50 mM Tris pH: 8, 1% NP-40, 0.5% Sodium Deoxycholate, 0.1% SDS) overnight (ON) at 4 °C. After centrifugation, supernatants were preserved, and protein concentration was measured using Bradford protein assay. Protein extracts were stored at −80 °C until its use. Equal amounts of protein were resolved by 0.1% SDS-10% polyacrylamide gel denaturing electrophoresis (SDS-PAGE) and transferred onto a nitrocellulose membrane. For LC3 proteins, we used a tricine SDS-PAGE with 16% polyacrylamide 6M urea gel [30]. The membranes were incubated with a specific anti-β-catenin, anti-p-Akt, anti-LC3-I, anti-LC3-II and anti-GAPDH antibodies ON at 4 °C, and then incubated with horseradish peroxidase-labeled secondary antibody for 1.5 h at RT. Finally, HRP chemiluminescence reaction was detected using a stable peroxide solution and an enhanced luminol solution. Images were obtained with an ImageQuant 4000 mini bioluminescent image analyzer (GE HealthCare LifeSciences, Marlborough, MA, USA) and analyzed using ImageJ 1.50b software package (National Institutes of Health, Bethesda, MD, USA).

### 2.10. Wound Healing Assay

MDA-MB-231 migration ability after transfection and EPI treatment was analyzed performing a wound healing assay. MDA-MB-231 cells (1 × 10^6^) were transfected as mentioned above. After EPI treatment, consistently shaped wounds were made using a sterile 100 µL pipette tip across each well, creating a cell-free area line [29,31]. Three images (r = 3) were captured in the same coordinates point at 0 h, 4 h, 8 h and 22 h after performing the wound. The experiment evaluates the same coordinates of each photo in different time points in order to evaluate migration ability. The gap size of the wounds was measured and analyzed using ImageJ 1.50b software package (National Institutes of Health, USA). The results were shown as free area of the wound, which is inversely proportional to the migration ability of the cells. The results were expressed as the decrease in the initial area of the wound, considering as 100% the area at time 0.

### 2.11. VEGF and FGF-2 ELISA

The secretion of specific pro-angiogenic factors was determined by ELISA. DuoSet hVEGF ELISA Kit (R&D System, Minneapolis, MN, USA) was used to detect human VEGF concentration from free-cell culture supernatants collected after treatments. FGF-2 expression levels were determined by DuoSet bFGF ELISA Kit (R&D System, USA) from protein extracts. The assays were carried out according to instructions provided by the manufacturer.

### 2.12. Particle Exclusion Assay

Variations in ECM after transfection with siUGDH and EPI treatment were analyzed through a particle exclusion assay [32]. MDA-MB-231 cells were plated in a 12 well-plate (3 × 10^4^) and transfected with UGDH siRNA or negative control siRNA, as described above. After 24 h, 1 µM EPI was added to complete 48 h of incubation after transfection. To determine the proportion of the pericellular area composed of HA, specific controls with active and heat-inactivated hyaluronidase from S. *hyaluroliticus* (SIGMA) were also performed during the assay, treating the tumor cells with two U/mL of hyaluronidase for 1 h. When treatments were completed, MDA-MB-231 cells were washed with PBS and 2 × 10^7^ fixed red blood cells were added to each well. After allowing red blood cells to decant for 30 min in an incubator, images of each condition were captured and analyzed using ImageJ 1.50b software package (National Institutes of Health, USA).

### 2.13. ELISA-Like Assay for Detection of Soluble HA

Since the synthesized HA can be secreted or can remain anchored to the cell membrane, it was decided to evaluate the concentration of this GAG in cell supernatants. The protocol was adapted from previous studies [33] and developed by our laboratory. A “sandwich” strategy was followed in which a specific HA-binding protein (HABP) was used to cover a 96-well plate. Once the samples of cell supernatants from the MDA-MB-231 cells were placed, HABP protein was added in its biotinylated form to determine the concentration of HA through the colorimetric detection of the peroxidase enzyme activity.

### 2.14. Confocal Microscopy for LC3 Subcellular Localization

MDA-MB-231 cells were plated on coverslips and co-transfected with UGDH or negative control siRNA plus two µg of EGFP-LC3 (# 11546, Addgene). 24 h after transfection, tumor cells were treated with 1 µM EPI as described above. After the treatment, the medium was removed, and cells were mounted on glass slides after being washed twice with PBS and fixed in 4% formaldehyde for 30 min. LC3 subcellular localization was analyzed by confocal microscopy using a Leica TCS SP5 instrument (Leica, Milan, Italy). The experiment was also performed treating each well with 20 µM chloroquine (an inhibitor of autophagy). Chloroquine was added to the cells concomitantly with EPI.

### 2.15. Patients and Samples for RT-qPCR

Four patients with breast cancer were selected for the analysis. The study included women over 18 years of age from the Surgery Department of Hospital Interzonal General de Agudos “Abraham Piñeyro” (HIGA) and Clínica Centro. The patients had previously signed an informed consent, approved (30.08.2018) by the ethics committee of the Hospital Austral, Province of Buenos Aires (17-006). This work has been carried out following The Code of Ethics of the World Medical Association. The investigations were carried out following the rules of the Declaration of Helsinki of 1975, revised in 2013.

Two types of samples were used: tumor tissue (TT) discarded at the time of the surgery and non-tumor tissue adjacent to the tumor (NAT). Tissue specimens were collected in the operation room and were evaluated by a pathologist. Selected patients did not previously receive antineoplastic treatment for the current disease. Patients with an advanced stage of cancer or metastasis were excluded from this study. The patients were all female (four patients) with mean age 61.50 ± 6.6 yr. Histopathologic diagnosis for all the breast cancer patients was invasive carcinoma of no special type (NST). TNM stages were determined by a pathologist and specific markers status such as prolactin receptor (PR), estrogen receptor (ER), HER2 and Ki67 were analyzed previously in our laboratory [34].

### 2.16. Tissue RNA Extraction and RT-qPCR

The tissue RNA was extracted using TRI reagent (Molecular Research Center, Inc., Cincinnati, OH, USA). A DNAse treatment was performed in order to degrade contaminating DNA and afterward reverse transcription with Oligo (dT) primers (Genbiotech) and M-MLV Reverse Transcriptase (M1701; Promega) to obtain cDNA. Taking into account that RNA is easily degraded, in order to preserve it, before extraction, a preservation solution RNAhold (TransGen Biotech Co, Beijing, China) was used. RNA yield was determined by Picodrop. To evaluate the expression of UGDH, previously prepared cDNA was amplified by real-time PCR using Universal SYBR Green Supermix (1725271, Bio-Rad Laboratories, Bio-Rad, Hercules, CA, USA) and 200nMol specific primers (Invitrogen, Life Technologies, ThermoFisher, Monza, Italy) for UGDH detection: Fw: 5′ GTGACTGAGAAAAGCACAGTTCC 3′ and Rv: 5′ CAGAAACTCAGGGTTGGACAG 3′. PCR conditions were 90 s at 94 °C and then 40 cycles of 30 s at 94 °C and 30 s at 60 °C. Relative levels of mRNAs were expressed as the “fold change” relative to the GAPDH gene (Fw: 5′ GGGGCTGCCCAGAACATCAT 3′ and Rv: 5′ GCCTGCTTCACCACCTTCTTG 3′). We used GAPDH as housekeeping gene considering that we never found much variability between our tumor samples. All determinations were performed as duplicates and a non-template control (NTC) was correspondingly added during every assay.

### 2.17. Kaplan–Meier Plots and TCGA Expression Data

To assess the prognostic value of UGDH and other functionally linked genes, the online tool kmplot.com [35], which allows a meta-analysis of gene expression in relation to breast cancer patient survival, was employed [36]. Gene expression data was obtained through microarray analysis of widely used arrays of the GEO database and converted into Kaplan–Meier plots. The package “survival” was used in the R programming (DataCamp, New York, NY, USA) environment to plot Kaplan–Meier survival curves and compute the number-at-risk [37]. To distinguish between high and low expression, the median was selected as cut off-value to reduce the impact of outliers and produce equal numbers in both categories to only show strong correlations. In addition, the JetSet probe set was selected to acquire unambiguous expression estimates [38] and redundant samples were removed to enhance the quality of the sample. Patients were stratified by ER, PR and HER2 status, analyzing only patients that had triple-negative tumors and received any chemotherapy treatment (*n* = 181). We analyzed the expression of UGDH, HASes, HYALs, pro-angiogenic factors (VEGF, EGF, FGF2), ABC pumps (ABCC1, ABCC2 and ABCG2), and autophagy markers (ATG16L1 and LC3-II). The correlation to survival was visualized by drawing Kaplan–Meier survival plots. The corresponding Affymetrix IDs are: 203342_at_UGDH; 207316_at_HAS1; 206432_at_HAS2; 223541_at_HAS3; 210619_at_HYAL1; 206855_at_HYAL2; 211728_at_HYAL3; 210512_at_VEGF; 204422_at_FGF2; 206254_at_EGF; 209735_at_ABCG2; 202804_at_ABCC1; 205887_at_ABCC2; 232612_at_ATG16L1 and 208786_at_LC3-II. The shown hazard ratios are not inverted (HR < 1 favorable).

**Figure 1:**
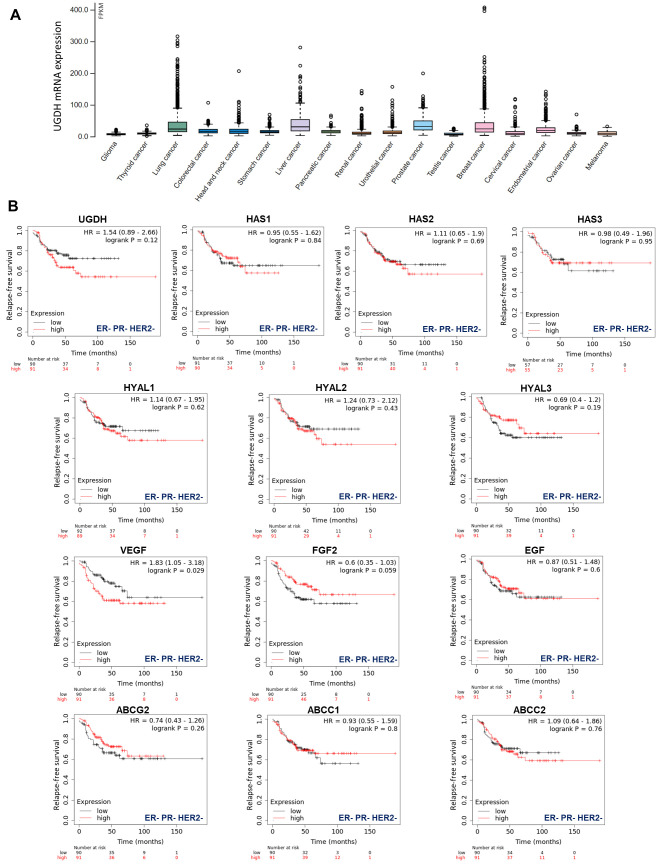
TCGA analysis of UGDH expression in different types of cancers. (**A**) Frequency of UGDH expression in different types of solid tumors. RNA-seq data of 17 cancer types are plotted as median number fragments per kb (FPKM) of exon per million reads generated by The Cancer Genome Atlas (TCGA). Points are displayed as outliers if they are above or below 1.5 times the interquartile range. The cancer types are color-coded according to which type of normal organ each cancer originated from. (**B**) The prognostic values of the expression of main genes involved in HA metabolism (UGDH, HA synthases and hyaluronidases), angiogenesis (VEGF, FGF2, EGF) and drug resistance (ABC drug transporters) in patients with breast cancer stratified by hormone receptor status Kaplan–Meier relapse-free survival curves are plotted based on triple-negative expression of ER, PR and Her2 treated with chemotherapy (*n* = 181). Log-rank p values and hazard ratios (HRs; 95% confidence interval in parentheses) are shown. The corresponding Affymetrix IDs are: 203342_at_UGDH; 207316_at_HAS1; 206432_at_HAS2; 223541_at_HAS3; 210619_at_HYAL1; 206855_at_HYAL2; 211728_at_HYAL3; 210512_at_VEGF; 204422_at_FGF2; 206254_at_EGF; 209735_at_ABCG2; 202804_at_ABCC1 and 205887_at_ABCC2.

Gene expression data, as plotted in Figure 1, were derived from The Pathology Atlas section [35] of the Human Protein Atlas (www.proteinatlas.org/) [38] using gene expression data of The Cancer Genome Atlas (TCGA, https://www.cancer.gov/tcga). RNA-seq data in 17 cancer types were reported as the median number of fragments per kilobase of exon per million reads. RNA cancer tissue category is calculated based on mRNA expression levels across all cancer tissues and included cancer tissue enriched, cancer group enriched, cancer tissue enhanced, expressed in all, mixed and not detected. Normal distribution across the dataset was visualized with box plots, shown as median, and 25th and 75th percentiles.

### 2.18. Protein Interaction Network Analysis

STRING v11 (http://string-db.org/) was used to generate in silico protein interaction networks for the gene products that were analyzed in Kaplan–Meier plots and carried out in experiments: UGDH, UGT2B7, HAS1, HAS2, HAS3, HYAL1, HYAL2, HYAL3, VEGF, EGF, PI3K, AKT,β-catenin, ABCC1, ABCC2, ABCG2, ATG16L1 and LC3-II. All interactions were predicted with a high confidence threshold and all active predictive methods were allowed. For the enrichment analysis, STRING implements well-known classification systems such as KEGG were used (Kyoto Encyclopedia of Genes and Genomes) [39].

### 2.19. Microarray Datasets Processing

In order to investigate the expression of UGDH in other breast cancer models, we decided to use publicly available microarray datasets from four cancer cell lines with different ER, PR and HER2 receptor status. Publicly available microarray dataset (GSE54326) was used for comparing differential UGDH expression levels in anthracycline-resistant breast cancer cells and control cells. NCBI GEO2R tool (http://www.ncbi.nlm.nih.gov/geo/geo2r/) was used to analyze UGDH mRNA expression levels [24].

### 2.20. Statistical Analysis

For statistical analysis, 95% confidence intervals (CI) were determined by calculating arithmetic mean values and variance (standard error of the mean, SEM) of three independent experiments. To evaluate if differences between the obtained values were significant: Student’s T test (T-test, Mann-Whitney) was used in the case of comparisons between two groups and analysis of variance (ANOVA, Tukey Test) was used to evaluate the differences between values of more than two experimental groups. The software Prism (GraphPad 5, San Diego, CA, USA) was used, considering a *p* value < 0.05 as statistically significant.

## 3. Results

### 3.1. The Expression of UGDH and Genes Associated with HA Metabolism, Angiogenesis and Drug Efflux in Breast Cancer Patients Stratified by Hormone Receptor Status

First, to have an overview of UGDH expression in different solid tumors, we analyzed its expression in samples from The Cancer Genome Atlas (TCGA), which demonstrated that UGDH is differentially expressed in lung, liver, prostate and breast cancers (Figure 1A).

Besides, our objective was to establish whether this enzyme could be proposed as a new biomarker of prognosis in breast cancer patients according to its hormone receptor status. We observed that higher levels of UGDH expression were correlated with a worse prognosis in patients with triple-negative breast cancer who had received chemotherapy (Kaplan–Meier plots, HR: 1.54 Figure 1B). We continued analyzing the possible associations between UGDH expression and the roles of different genes related to tumor progression for the survival prognosis of breast cancer patients by analyzing Kaplan–Meier plots. Specifically, we studied genes involved in drug resistance, such as ATP-binding cassette (ABC) drug transporters, and genes that codify pro-angiogenic factors and enzymes involved in HA metabolism. It was shown that increases in the expression of HAS2, HYAL1-2, VEGF and ABCC2 were related to worse prognosis in patients with triple-negative breast cancer (HR: 1.11, 1.14, 1.24, 1.83 and 1.09 respectively, Figure 1B). On the other hand, the rest of genes analyzed (HAS1-3; HYAL3; FGF2; EGF; ABCG2; ABCCC1) did not show any marked effect on patient survival, or had some protective effect on it (Figure 1B).

**Figure 2:**
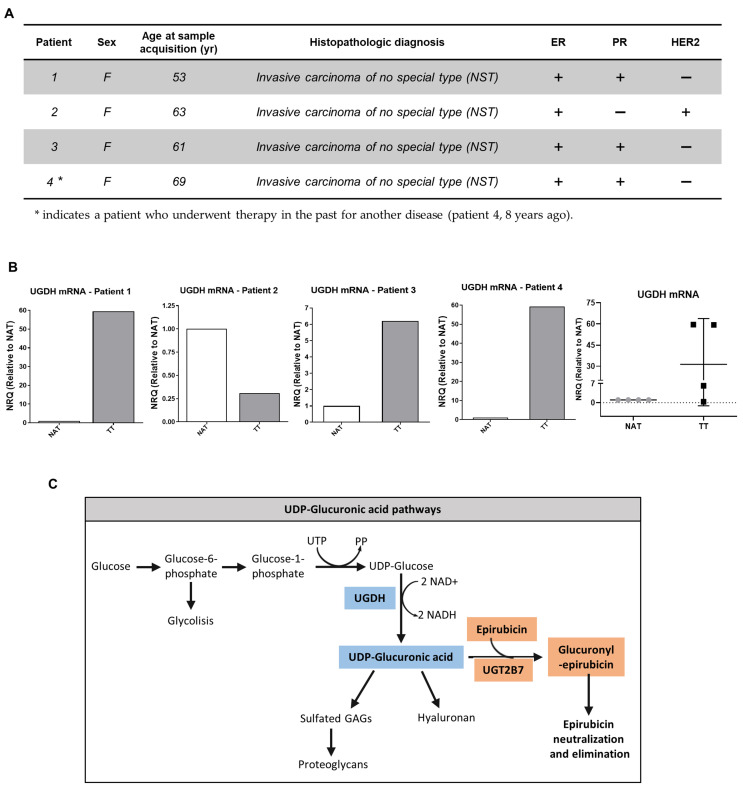
(**A**,**B**). Real-time PCR analysis of UGDH expression in patients with breast cancer. UGDH mRNA levels were normalized to housekeeping gene GAPDH and shown as tumor tissue (TT) relative to non-tumor tissue adjacent to tumor (NAT) of four different patients with breast cancer, characterized according to clinical parameters and ER, PR and HER2 status. (**C**). Schematic illustration of UDP-GlcUA pathways: GAG and PG synthesis, and role in EPI elimination.

Furthermore, in the present study we analyzed the expression of UGDH mRNA in samples obtained from tumors and adjacent normal tissue of breast cancer patients previously characterized according to ER, PR, HER2 and Ki67 status in our laboratory (Figure 2A) [32]. For this purpose, tissues obtained from surgical patients were processed; mRNA was extracted and analyzed as described above. All of the results were shown as tumor tissue (TT) relative to non-tumor tissue adjacent to tumor (NAT). In patients 1, 3 and 4, who were defined as HER2− patients (Figure 2A); we observed an increase in UGDH expression in TT compared to NAT.

On the other hand, patient number 2, who was defined as PR− and HER2+ (Figure 2A); showed a decrease in UGDH levels in TT respect to NAT (Figure 2B), which was also related to low HA and HAS-2 levels, as was previously observed (see [32]).

The results found in both in silico and breast cancer patient studies allowed us to hypothesize that the UGDH enzyme is involved in the progression of triple-negative breast cancer, even in cases that have received chemotherapeutic treatment. For this reason, we decided to continue studying the mechanisms involved in the appearance of resistance to EPI in which UGDH and UDP-GlcUA could be involved. Figure 2C summarizes the pathways involved in the detoxification of EPI and in the synthesis of HA and PGs, where UGDH is a key point of connection within both processes, supporting our hypothesis.

### 3.2. Analysis of UGDH Expression and Cell Integrity after UGDH Knockdown and EPI Treatment in the MDA-MB-231 Breast Cancer Cell Line

**Figure 3:**
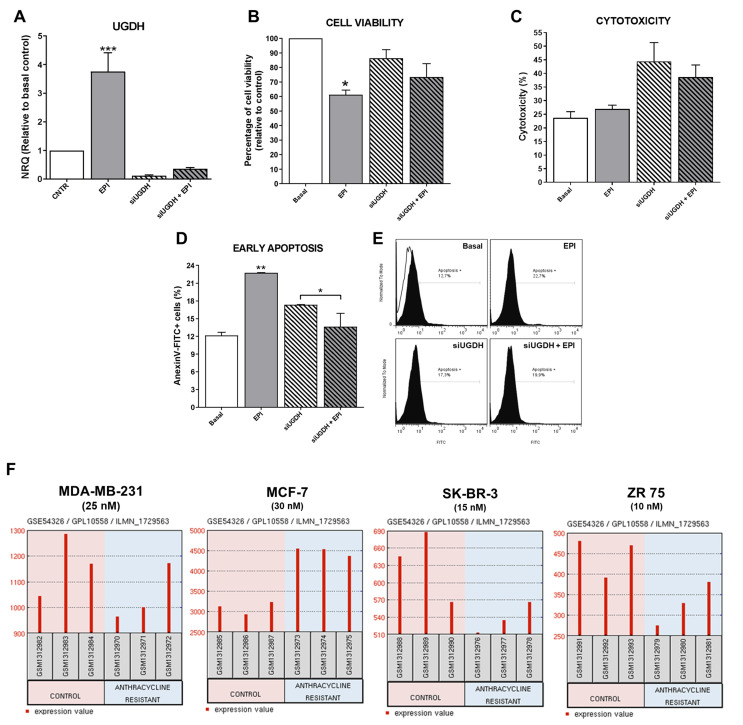
Analysis of MDA-MB-231 cells after silencing of UGDH and EPI treatment. MDA-MB-231 cells were transfected with a specific siRNA against UGDH gene (siUGDH) using a random sequence siRNA as negative control (siSCR). After 24 h, 1 µM EPI (1 EPI) was added to complete 48 h of incubation. UGDH mRNA levels were obtained by real-time quantitative PCR using Taqman probes (**A**). Cell viability was measured performing a MTT assay (**B**) and cytotoxicity was determined evaluating the activity of lactate dehydrogenase (LDH) enzyme in cell supernatants (**C**). Early apoptosis (**D**,**E**) was detected by flow cytometry using AnnexinV-FITC stain. Histograms (**E**) show the most representative of three independent experiments performed with 50,000 events/condition. *n* = 3, mean ± SEM, * *p* < 0.05, ** *p* < 0.01, *** *p* < 0.001. (**F**) Microarray dataset GSE54326 specific for different breast cancer cell lines was used for comparing differential UGDH expression levels in tumor cells resistant to anthracyclines treatment and control cells. In each case, NCBI GEO2R tool was used to analyze UGDH levels.

First, to verify that our transfection system worked correctly, it was decided to analyze the expression levels of UGDH after completing the transfection scheme and post-treatment with EPI. As expected, after 48 h, MDA-MB-231 cells transfected with UGDH-specific siRNA showed a significant reduction in UGDH expression level (siUGDH, 90% respect to control). Surprisingly, we found a significant increase in the expression of UGDH in response to the treatment with 1 µM EPI (EPI) (*** *p* < 0.001). However, the silencing of UGDH combined with EPI treatment (siUGDH + EPI) produced a significant reduction of UGDH mRNA levels with respect to EPI (Figure 3A).

An important consideration during transfection experiments is to monitor possible alterations in cell integrity during cell culture because of nucleofection and antitumor treatment with EPI. For that reason, we decided to evaluate the effects of both transfection and EPI treatment on cell viability, cytotoxicity and apoptosis.

As expected, only the treatment with EPI decreased cell viability 40%, since we used a concentration close to the IC_50_ value (IC_50_ MDA-MB-231: 4.9 µM) in order to obtain viable cells for subsequent assays (* *p* < 0.05), as was determined previously [26]. A decrease in cell viability was found when comparing the condition of transfections with siUGDH and siSCR (data not shown). Although a slight accentuation of this effect was observed in the presence of EPI (siUGDH + EPI and siSCR + EPI respectively), these differences were not statistically significant, and the levels remained above the values of treatment with 1 µM EPI (Figure 3B). The cell viability of the siSCR control did not differ significantly from the basal control (data not shown).

When cytotoxicity was evaluated by LDH release, we observed increased levels in all conditions in which cells were transfected. However, the obtained results were not statistically significant among different conditions (Figure 3C). The slight effect observed on cytotoxicity was within expected range since the transfection method involves the electroporation of the cell membrane that can release LDH enzyme to the culture medium. Finally, we found unexpected results when analyzing the induction of apoptosis after transfection and EPI treatment. We observed higher levels of apoptosis after EPI treatment compared to basal conditions (EPI vs. basal ** *p* < 0.01). However, apoptosis induction after silencing of UGDH and EPI treatment was significantly reduced compared with transfected cells without EPI treatment (siUGDH + EPI vs. siUGDH * *p* < 0.05). Nevertheless, apoptosis levels in those conditions (siUGDH + EPI) remained below the levels obtained with 1µM EPI treatment (EPI) (Figure 3D,E). Therefore, the knockdown of UGDH during EPI treatment rescues MDA-MB 231 cells from the apoptosis induction caused by EPI.

To complement in vitro experiments on MDA-MB-231 cells, GEO2R (https://www.ncbi.nlm.nih.gov/geo/geo2r/) was applied to screen how UGDH mRNAs are expressed in widely used breast cancer cell lines with different aggressive phenotypes and hormone-receptors status. In this analysis, we included results from public databases of breast cancer cell lines (MDA-MB-231, MCF-7, SK-BR-3 and ZR-75) treated or not treated with anthracyclines, under treatment conditions similar to those carried out in our work. As mentioned above, MDA-MB-231 cells are one of the most used models of triple negative breast cancer, with a basal phenotype and metastatic characteristics. MCF-7 is a poorly aggressive, non-invasive and ER+ and PR+ cell line. SK-BR-3 cell line overexpresses the HER2 (Neu/ErbB-2) gene product and ZR-75 cells are considered ER+, PR+ and HER2+, with a luminal phenotype [28].

In this analysis, we found higher levels of UGDH gene in MDA-MB-231 and MCF-7 cell lines compared to the other cell lines (Figure 3F). This result could be related to the fact that these two cell lines are HER2−. Similar results were found when evaluating the expression of UGDH in patients with breast cancer, according to HER2 status (Figure 2). Furthermore, we analyzed the expression of UGDH when the different cell lines were exposed to different EPI concentrations (nM) in order to obtain resistant clones. In response to EPI, breast cancer cells altered the expression of UGDH, which indicates that it could act as a marker associated with this type of cancer. Specifically, we detected a significant increase in UGDH levels when comparing resistant and non-resistant phenotypes of MCF-7 cell line, and in MDA-MB-231 higher expression was observed in control conditions (Figure 3F).

### 3.3. Evaluation of Intracellular Accumulation of EPI after Knockdown of UGDH Enzyme

EPI is a molecule capable of emitting fluorescence in the red spectrum, which is detectable in flow cytometry assays (excitation peak 480 nm and emission peak 590 nm). This allowed the intracellular analysis of EPI accumulation in MDA-MB-231 cells after transfection of siUGDH or siSCR, evidencing the appearance of positive fluorescence in this spectrum.

**Figure 4:**
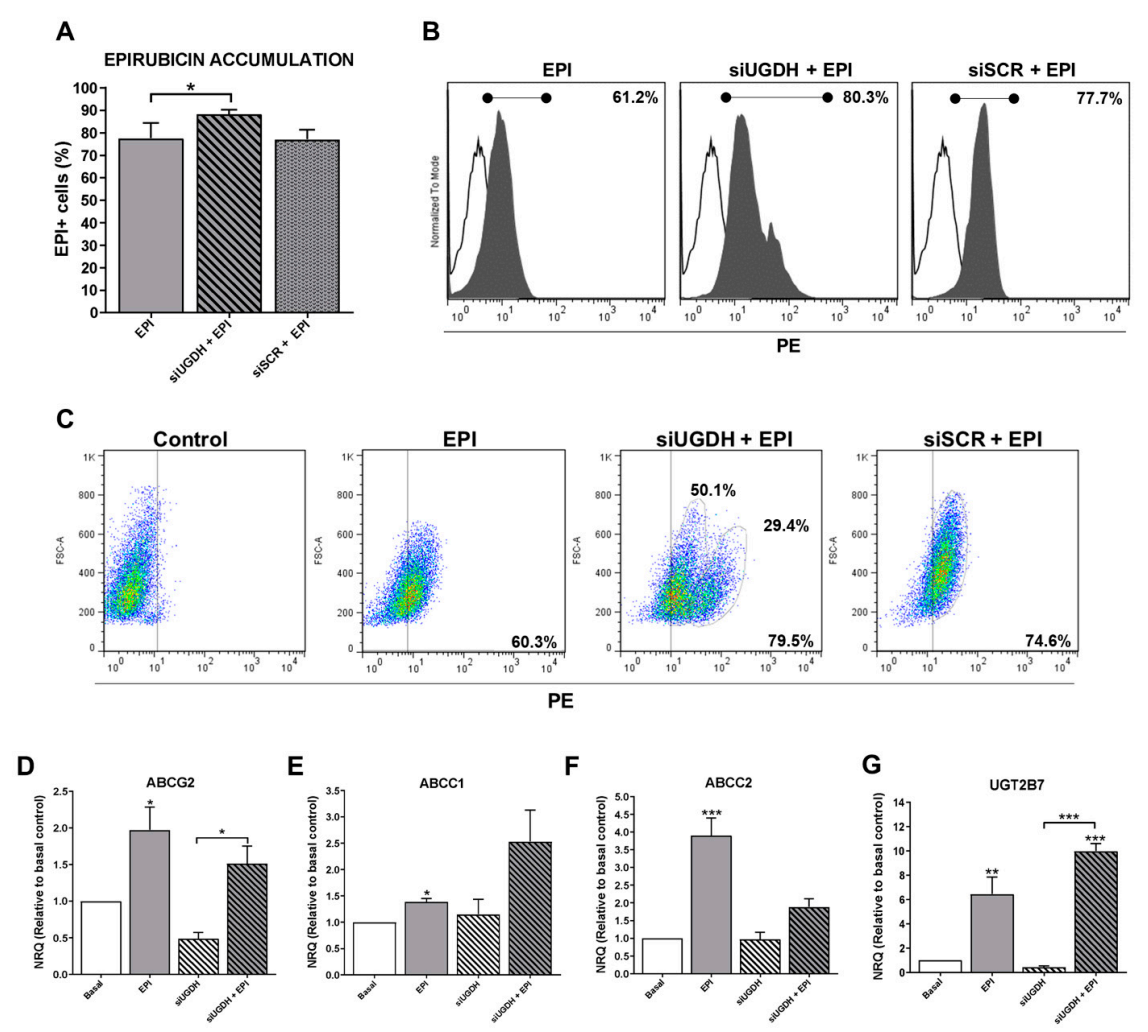
Evaluation of intracellular EPI accumulation in MDA-MB-231 cells after knockdown of UGDH gene. MDA-MB-231 cells were transfected with a specific siRNA against UGDH gene (siUGDH) using a random sequence siRNA as the negative control (siSCR). After 24 h, 1 µM EPI (EPI) was added to complete 48 h of incubation. Intracellular EPI accumulation was measured by flow cytometry. A control with MDA-MB-231 cells treated only with EPI (EPI) was added to determine the basal uptake of the drug. Bars show the percentage of EPI+ cells determined by comparison with a negative control (**A**). Histograms (**B**) and dot plots (**C**) show the most representative of four independent experiments performed with 50,000 events/condition. *n* = 4, mean ± SEM, * *p* < 0.05. To evaluate the expressions of ABCG2 (**D**), ABCC1 (**E**), ABCC2 (**F**), UGT2B7 (**G**), drug efflux pumps and the specific EPI transferase UGT2B7, total RNA was extracted and 2 µg was reverse transcribed by RT-PCR. cDNAs were subjected to real-time quantitative PCR using SYBR Green. Results were normalized using β-actin as reference gene and all determinations were performed as duplicates. *n* = 3, mean ± SEM, * *p* < 0.05 ** *p* < 0.01 *** *p* < 0.001.

We observed higher intracellular EPI accumulation in tumor cells that had been transfected with siUGDH compared with non-transfected cells (siUGDH + EPI vs. EPI * *p* < 0.05) (Figure 4A,B). Moreover, two well-differentiated populations of MDA-MB-231 cells in terms of EPI accumulation were observed only when cells were transfected with siUGDH before being treated with EPI (siUGDH + EPI), as could be observed in the flow cytometry analysis. These results were not found in the treatments carried out with EPI (EPI) or in MDA-MB-231 cells transfected with siSCR (siSCR + EPI) (Figure 4C). These results indicated that there are subpopulations of MDA-MB-231 cells capable of responding differentially to EPI, favoring less drug accumulation and avoiding apoptosis. We hypothesized that this effect is caused by the intrinsic heterogeneity of the MDA-MB-231 cell line and its aggressive phenotype. Besides, we ruled out that it was an effect of the transfection system since the control with siSCR did not give the same results. Considering these previous results, we decided to continue evaluating possible mechanisms involved in the development of drug resistance.

### 3.4. Modulation of Expression of ABC Drug Efflux Transporter Genes and Their Inactivation after UGDH Knockdown and EPI Treatment

To understand possible mechanisms that involve the evasion of EPI anti-tumoral effects despite its higher and differential cell accumulation, we analyzed the expression of different genes related to the drug inactivation.

Since the silencing of UGDH affected the intracellular accumulation of EPI, this could be related to possible changes in the expression of drug efflux pumps implicated in EPI elimination. For that reason, we started analyzing the expression of different ABC drug transporters implicated in EPI efflux: ABCG2, ABCC1 and ABCC2. We found a significant increase in mRNA levels in response to EPI treatment compared with basal conditions (EPI vs. basal: ABCG2 and ABCC1 * *p* < 0.05; ABCC2 ** *p* < 0.01). Nevertheless, the silencing of UGDH (siUGDH) and the combination with EPI treatment (siUGDH + EPI) did not induce any significant modulation of their expression compared to basal conditions or EPI treatment (Figure 4D,E,F). These data could explain the increased intracellular accumulation of EPI after siUGDH transfection. However, it is controversial regarding the results observed for the apoptosis process, indicating that an increase in EPI intracellular accumulation is not directly associated with an increase in cell death.

On the other hand, the UGT2B7 enzyme is the exclusive transferase responsible for binding EPI to UDP-GlcUA (Figura 2C). As previously reported [15], after EPI treatment we found a significant upregulation in the expression of the UGT2B7 compared to basal conditions (EPI vs. basal ** *p* < 0.01) (Figure 4G). Even more, we observed increased levels in UGT2B7 expression when breast cancer cells were transfected with siUGDH before treating them with EPI (siUGDH + EPI) (Figure 4G). This result indicated that, although EPI efflux was not completely activated, tumor cells upregulated the expression of this enzyme in response to EPI treatment, as a new mechanism of drug resistance through its inactivation by glucuronidation.

### 3.5. Effects of UGDH Knockdown and EPI Treatment on Tumor Angiogenesis, Cell Proliferation and Migration

**Figure 5:**
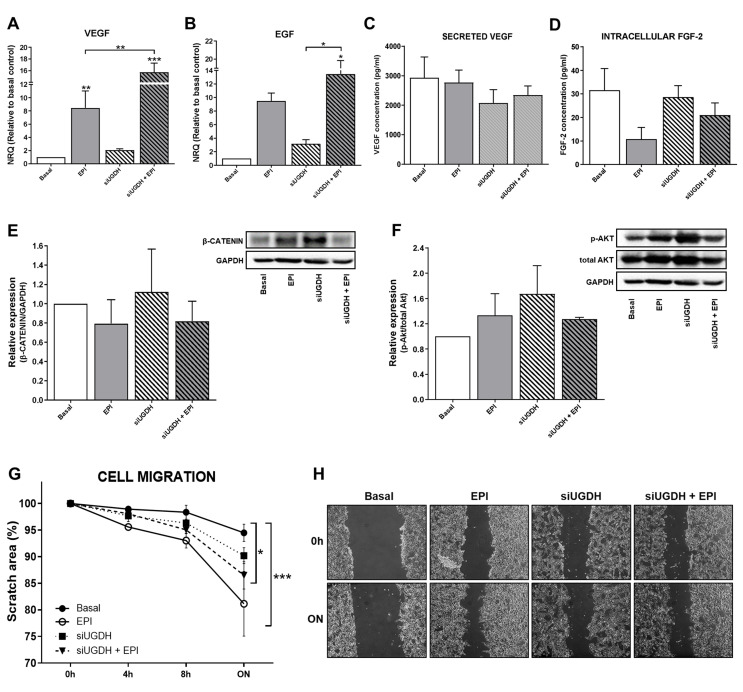
Evaluation of angiogenic response, cell proliferation and migration after silencing UGDH and EPI treatment. MDA-MB-231 cells were transfected with a specific siRNA against UGDH gene (siUGDH) using a random sequence siRNA as the negative control (siSCR). After 24h, 1 µM EPI (1 EPI) was added to complete 48 h of incubation. VEGF (**A**) and EGF (**B**) expressions were obtained by real-time quantitative PCR using SYBR Green. Results were normalized using β-actin as the reference gene and all determinations were performed as duplicates. Levels of secreted VEGF in cell supernatants (**C**) and intracellular levels of FGF-2 in cell lysates (**D**) were measured by ELISA. Total protein was extracted and the expression of β-catenin (**E**) and p-AKT (**F**) were detected by Western blot. The images show the most representative of the three independent experiments. To describe cell migration ability, consistently shaped wounds were made during transfection and EPI treatment. The experiment evaluated the same coordinates of each photo at different time points in order to evaluate migration ability. Three images were captured at 0 h, 4, 8 and 22 h at the same coordinates. The gap sizes of the wounds were measured and analyzed using ImageJ software. The results were shown as free area of the wound, which is inversely proportional to the migration ability of the cells. The results were expressed as the decrease in the initial area of the wound, considering as 100% the area at time 0 (**G**). Micrographs show the most representative of three independent experiments (**H**). *n* = 3, as mean ± SEM. * *p* < 0.05 ** *p* < 0.01 *** *p* < 0.001.

Among the mechanisms involved in drug resistance, we have previously reported that during chemotherapy treatment tumor cells can modulate angiogenesis by altering the behavior of endothelial cells. Tumor cells can secrete different pro-angiogenic factors, such as VEGF, FGF-2 and EGF, among others [40,41,42], capable of promoting the migration of endothelial cells and the formation of blood vessels. For that reason, we analyzed the mRNA expression levels of VEGF and EGF after UGDH knockdown and EPI treatment. We observed a significant increase in the expression of VEGF after treating tumor cells with EPI (EPI vs. basal ** *p* < 0.01 Figure 5A). In turn, we observed even higher levels of VEGF mRNA when MDA-MB-231 cells were first transfected with UGDH siRNA and treated with EPI (EPI vs. siUGDH + EPI ** *p* < 0.01 Figure 5A). In the case of EGF, we found a similar tendency of upregulation in response to EPI treatment; however, we only obtained statistically significant differences when cells were transfected with siUGDH and treated with EPI with respect to transfected cells non-treated with EPI (siUGDH + EPI vs. siUGDH * *p* < 0.05 Figure 5B).

Afterward, we analyzed the biosynthesis and secretion levels of VEGF by ELISA. We did not observe significant changes in VEGF concentration in supernatants from MDA-MB-231 cells transfected with siUGDH + EPI (Figure 5C). FGF-2 is a potent cell survival factor involved in tumor angiogenesis [43,44]. Thus, we decided to evaluate FGF-2 biosynthesis. First, we analyzed FGF-2 protein expression by using supernatants from MDA-MB-231 that have been transfected with siUGDH and treated with EPI. However, no detectable levels were found by ELISA (data not shown).

Since FGF-2 is not frequently detected in the media of cultured cells—because it remains associated with cell-surface heparan sulfate proteoglycans upon secretion [45,46,47]—we performed an ELISA with total cellular protein extract and we were able to detect it. However, we did not find significant differences between treatment and basal conditions at the time of the assay (Figure 5D). These results could be associated with the fact that the samples for protein analysis were collected at the same time as those for mRNA analysis. That did not allow us to detect significant changes at the translational level.

On the other hand, tumor cells can avoid anti-tumoral treatment and generate drug resistance by the modulation of specific signaling pathways related to cell survival and proliferation. In this sense, we decided to analyze possible modulation in Wnt/β-catenin and PI3K/Akt pathways after the UGDH knockdown and EPI treatment. The expression of β-catenin and p-Akt proteins was analyzed by Western blot from total protein extracts. When β-catenin was evaluated, we observed a tendency to increase the expression levels in response to the silencing of the UGDH enzyme. However, we did not find significant differences in comparison to basal conditions or EPI treatment (Figure 5E). Finally, when we studied the expression of the active form of Akt (p-Akt), we did not find significant differences between treatments (Figure 5F).

Subsequently, the migration ability of tumor cells has been determined as another process that indicates drug resistance and their ability to spread during chemotherapy. We observed that in response to EPI treatment, MDA-MB-231 cells increased their migration compared to basal conditions (EPI vs. basal *** *p* < 0.001) (Figure 5G,H). The same effect was observed when we analyzed the silencing of UGDH and EPI treatment (siUGDH + EPI vs. basal * *p* < 0.05) (Figure 5G,H). These results indicate that, contrary to expectations, EPI enhances tumor cell migration even under conditions where UGDH is inhibited, beyond its glucuronidation status. These findings are in concordance with the aggressive features of this cell line and their capacity to develop EPI resistance.

### 3.6. The Effect of UGDH Knockdown and EPI Treatment on Autophagy

**Figure 6:**
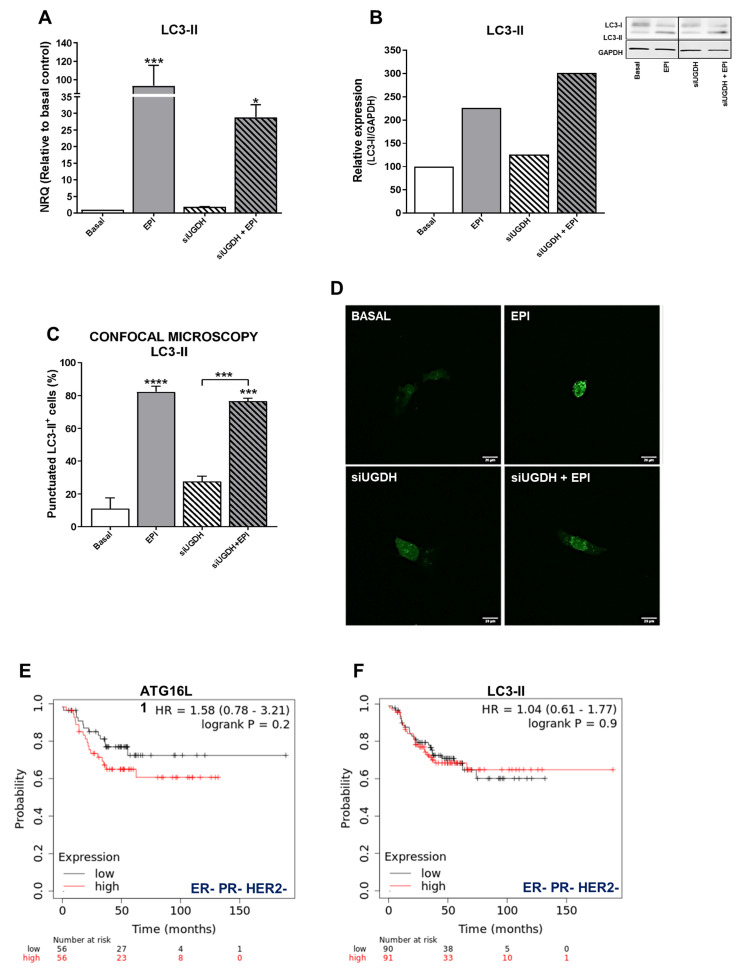
Analysis of autophagy as a mechanism involved in drug resistance. MDA-MB-231 cells were co-transfected with a specific siRNA against UGDH gene (siUGDH) or a random sequence siRNA as negative control (siSCR) and 2 µg of a specific LC3-GFP construct. After 24 h, 1 µM EPI (1 EPI) was added to complete 48 h of incubation. LC3-II expression (**A**) was determined by real-time quantitative PCR using SYBR Green. Results were normalized using β-actin as the reference gene and all determinations were performed as duplicates. Transformation of LC3-I in LC3-II was evaluated by Western blot, comparing both with GAPDH reference protein (**B**). To analyze the formation of autophagosomes, the fluorescence emitted by GFP was evaluated through confocal microscopy (**D**). The LC3-II puncta analysis was performed using the ImageJ software (**C**). *n =* 3, mean ± SEM, * *p* < 0.05 *** *p* < 0.001, **** *p* < 0.0001. The prognostic value of the expression of ATG16L1 (**E**) and LC3-II (**F**) in patients with breast cancer stratified by hormone receptor status. Kaplan–Meier relapse-free survival curves are plotted based on triple-negative expression of ER, PR, and HER2 treated with chemotherapy (*n* = 181). Log-rank p values and hazard ratios (HRs; 95% confidence interval in parentheses) are shown. The corresponding Affymetrix IDs are: 232612_at_ATG16L1 and 208786_at_LC3-II.

Several studies have shown that autophagy can protect cancer cells from death induced by anti-tumoral drugs, so autophagy might be related to the development of drug resistance to these agents [46]. Since we observed that despite the induction of intracellular accumulation of EPI after UGDH knockdown, it failed to increase apoptosis, we decided to study the modulation of autophagy as a possible mechanism involved in EPI resistance in breast cancer cells transfected with UGDH siRNA. First, we evaluated the mRNA expression of the autophagosome marker, LC3-II (Figure 6A). We observed an upregulation of LC3-II levels in response to EPI treatment compared with basal conditions (EPI vs. basal *** *p* < 0.001). We also found an increase in LC3-II expression when MDA-MB-231 cells were transfected with UGDH siRNA and after they were treated with EPI, compared to UGDH siRNA alone (Figure 6A).

Since the differences found in the expression of LC3-II indicated a possible positive modulation of autophagy in response to EPI treatment, we decided to continue analyzing the formation of autophagosomes in tumor cells after UGDH silencing and treatment with EPI. A co-transfection system with the specific siRNAs (UGDH or SCR) was carried out together with the expression vector of a fusion protein LC3-II-GFP (green fluorescent protein) (Figure 6C,D). In line with previous results, we detected an increase in the formation of autophagosomes in response to antitumor treatment with EPI compared with basal control (EPI vs. basal **** *p* < 0.0001). Moreover, we found increased levels of LC3-II-positive autophagosomes in MDA-MB-231 cells that had first been silenced regarding UGDH, and after that treated with EPI. These results were confirmed through the analysis of the protein expression of LC3-I and LC3-II by Western blot. We found that LC3-II protein levels were even higher when the UGDH enzyme was silenced before treating tumor cells with EPI (Figure 6B). Taken together, these results would indicate that breast cancer cells respond to EPI treatment by favoring tumor survival and adaptation to the stress generated by the antitumor treatment. Moreover, we observed that the lack of UGDH could support the development of resistance to EPI through the process of autophagy. Both the Western blot and the GFP-LC3 experiments were performed using chloroquine, a specific inhibitor of autophagy, as a negative control (Supplementary Figure S1).

In order to establish whether the expression of autophagy markers could have an impact on breast cancer prognosis, we investigated their relation to relapse-free survival (RFS) in patients with ER– PR− and HER2− tumors (Figure 6E,F). Gene expression data from tumor samples of triple-negative cancer patients that received chemotherapy (*n* = 181) were analyzed using the online tool Kaplan–Meier Plotter [35]. Specifically, we observed that the expression of specific autophagy markers such as ATG16L1 and LC3-B was associated with poor relapse-free survival (HR 1.58 and 1.04, Figure 6E,F).

### 3.7. Effects of UGDH Knockdown and EPI Treatment on Extracellular Matrix and HA Expression

**Figure 7:**
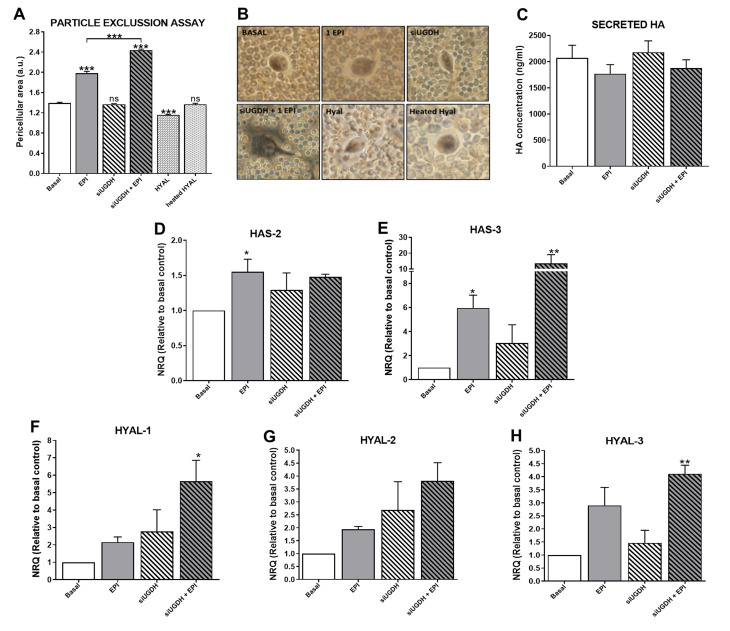
Evaluation of ECM variations as a consequence of silencing of UGDH and EPI treatment. MDA-MB-231 cells were transfected with a specific siRNA against UGDH (siUGDH) and a random sequence siRNA as the negative control (siSCR). After 24h, 1 µM EPI (1 EPI) was added to complete 48 h of incubation. To perform the particle exclusion assay, 2 × 10^7^ fixed red blood cells were added to each well. After allowing one to decant, multiple images were captured and analyzed using ImageJ software (**A**). Micrographs show the most representative of three independent experiments (**B**). Secreted HA in cell supernatants was measured by ELISA (**C**). HAS2 (**D**), HAS3 (**E**), HYAL-1 (**F**), HYAL-2 (**G**) and HYAL-3 (**H**) expressions were obtained by real-time quantitative PCR using SYBR Green or Taqman probes. Results were normalized using β-actin as the reference gene and all determinations were performed as duplicates. *n* = 3, mean ± SEM, * *p* < 0.05 ** *p* < 0.01 *** *p* < 0.001.

We continued studying the effects of UGDH knockdown and EPI treatment on the tumoral ECM and its components, such as HA. It is well known that tumor ECM modulates the mechanism of drug resistance [4,22,26]. Besides, UDP-GlcUA is a precursor for the synthesis of several GAGs and PGs (Figure 2C), and the silencing of UGDH can modulate its production [9]. To evaluate the ability of tumor cells to generate an interstitial or pericellular matrix, a particle exclusion assay was performed in MDA-MB-231 cells during UGDH siRNA transfection and EPI treatment. Surprisingly, we observed that the pericellular area of MDA-MB-231 cells remained similar to basal conditions when the UGDH enzyme was silenced (Figure 7A,B). When tumor cells were treated only with EPI as a control, a significant increase in the pericellular area was observed in comparison to basal conditions (EPI vs. basal *** *p* < 0.001).

This effect was also observed when tumor cells were treated with EPI after UGDH knockdown, compared to the effect produced by EPI (siUGDH + EPI vs. EPI *** *p* < 0.001) (Figure 7A,B).The inclusion of a specific control with a hyaluronidase enzyme during the assay (HYAL) allowed us to estimate which fraction of this pericellular matrix is composed of HA. In this case, when tumor cells were treated with HYAL before the addition of the red blood cells, a small pericellular area was observed, with a statistically significant decrease with respect to basal conditions (HYAL vs. basal *** *p* < 0.001) (Figure 7A,B). All these data indicate that after UGDH knockdown, although MDA-MB-231 cells had a diminished availability of UGDH enzyme to synthesize UDP-GlcUA, they were able to favor the synthesis of GAGs within the ECM components in this condition. Moreover, it was demonstrated that the pericellular matrix of these tumor cells was mainly composed of HA.

Moreover, it is known that not all synthesized HA remains in the plasma membrane. HA chains can be released to cell media during the culture of tumor cells. To determine the levels of HA that were secreted to the cell medium, we performed an ELISA like-assay using a specific HA binding protein (HABP). In this case, we did not observe significant differences in HA secretion under UGDH knockdown or EPI treatment (Figure 7C).

### 3.8. Modulation of HA Metabolism: A Balance between HASes and HYALs

To continue analyzing the modulation of HA synthesis, under the silencing of UGDH and treatment with EPI, we decided to investigate a possible association between the results previously obtained and HA, regarding the modulation of its metabolic enzymes.

For that reason, we evaluated the mRNA expression of the (i) synthesizing enzymes of HA—HAS2 (Figure 7D) and HAS3 (Figure 7E); and (ii) HA degrading enzymes—HYAL1 (Figure 7F), HYAL2 (Figure 7G) and HYAL3 (Figure 7H). When we analyzed the expression levels of HAS2 and HAS3, we observed an increase in the expression of both enzymes in response to EPI treatment (EPI vs. basal * *p* < 0.05) (Figure 7D,E). In turn, the silencing of UGDH enzyme combined with EPI treatment further increased the expression levels of both enzymes in comparison with basal control (Figure 7D,E).

Furthermore, the expression levels of the main HA degrading enzymes were analyzed. In all cases, we observed a similar pattern of increase in the expression of the three enzymes, where EPI per se was able to upregulate the expression of the three HYALs compared to basal conditions (Figure 7F–H). Furthermore, we found even higher expression levels of HYALs when MDA-MB-231 cells were first transfected with siUGDH and subsequently treated with EPI (Figure 7F–H). Taking these results together, we can conclude that even under the silencing of the enzyme involved in HA synthesis and with an anti-tumoral treatment such as EPI, MDA-MB-231 breast cancer cells were able to favor the synthesis of this GAG. Even more, tumor cells responded to both conditions by increasing ECM deposition as another mechanism of drug resistance.

### 3.9. Functional Enrichment Analysis for UGDH-Related Proteins

**Figure 8:**
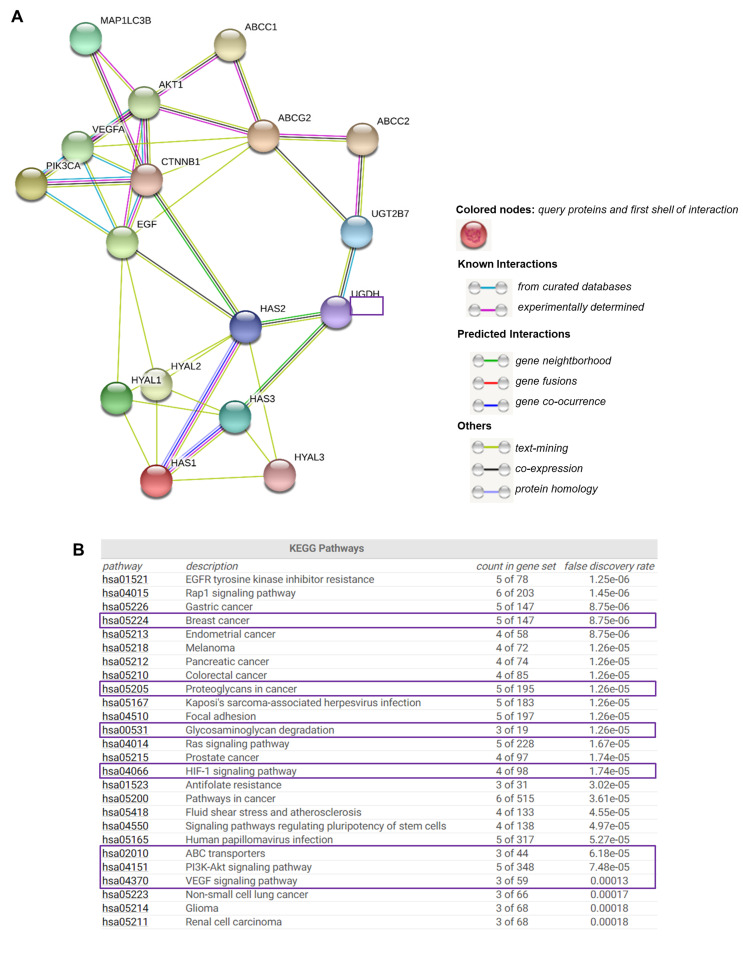
The complex network of genes involved in drug resistance, hyaluronan metabolism and angiogenesis. STRING database output depicting functional and physical interactors of UGDH, UGT2B7, HAS1, HAS2, HAS3, HYAL1, HYAL2, HYAL3, PI3K (PIK3CA), AKT1, b-CATENIN (CTNNB1), VEGF, EGF, LC3-II (MAP1LC3B), ABCC1, ABCC2 and ABCG2 obtained from http://string-db.org/(**A**). KEGG pathway analysis (**B**).

Due to understanding the importance of analyzing the protein-level functionality of UGDH and its possible interactions with other cell effectors, we used the online bioinformatics tool STRING [39] to find functional interaction networks of UGDH enzyme and its related genes and proteins analyzed in vitro up to this point in our results. STRING tool generated in silico protein interaction networks for the gene products that we analyzed in Kaplan–Meier plots and carried out experiments with: UGDH, UGT2B7, HAS1, HAS2, HAS3, HYAL1, HYAL2, HYAL3, VEGF, EGF, PI3K, AKT, β-catenin, ABCC1, ABCC2, ABCG2, ATG16L1 and LC3-II. The STRING analysis showed that UGDH has close interactions only with UGT2B7, HAS2 and HAS3, indicating that it is directly involved in HA synthesis and glucuronidation reactions. Indeed, UGT transferase interacts with the studied drug efflux pumps, showing a relation between the responses to chemotherapy that requires glucuronidation and drug efflux (Figure 8A). As expected, the enzymes related to HA metabolism (HASes and HYALs) were interrelated with each other, showing a strict regulation. The rest of the molecules are also interconnected. Besides, we observed that both PI3K/AKT and Wnt signaling pathways serve as links between the mechanisms evaluated in the present work: autophagy, angiogenesis, drug resistance and HA metabolism.

STRING implements well-known classification systems such as KEGG (Kyoto Encyclopedia of Genes and Genomes). This tool allowed us to statistically analyze and compare the different cellular processes in which STRING-analyzed protein effectors are involved. Interestingly, the KEGG analysis allowed us to identify enriched pathways linked to highly aggressive types of cancer, such as angiogenesis (HIF-1 and VEGF, PI3K/Akt and the differential expression of PGs and GAGs, and ABC drug transporters (Figure 8B).

## 4. Discussion

EPI is considered one of the most active drugs used in the treatment of breast cancer resistant to hormonal therapy or triple-negative breast cancer [14]. EPI produces similar efficacy as DOX with less adverse effects, due to a differential elimination mechanism through a 4-O-glucuronidation reaction [48,49]. This reaction occurs mainly in the liver, where the enzyme UGT2B7 transfers a molecule of UDP-GlcUA to EPI [17,20]. It has been shown that, in hepatocellular carcinoma cells, the expression of this enzyme is tightly regulated during EPI treatment through the p53 pathway. In turn, different studies have analyzed the role of UGDH as a marker of tumor progression during chemotherapy with drugs that are eliminated by glucuronidation. On the other hand, it generates the UDP-GlcUA substrate hence the activity of these enzymes is related to glucose metabolism and the synthesis of GAGs and PGs [50,51]. UGDH enzyme transforms UDP-glucose (UDP-Glc) into UDP-GlcUA, a substrate of the specific enzymes that synthesize HA [6]. As we showed in the schema of Figure 2, the EPI inactivation or resistance could be associated with GAGs metabolism in cancer cells.

The UGDH knockdown strategies have been proposed to evaluate the role of a potential modulator of breast cancer behavior [52], considering that this reduces the intracellular UDP-GlcUA availability and therefore modulation of ECM composition at PGs and GAGs levels, which are implicated in tumor progression. Therefore, this strategy could affect the responses to different tumor therapies.

**Figure 9:**
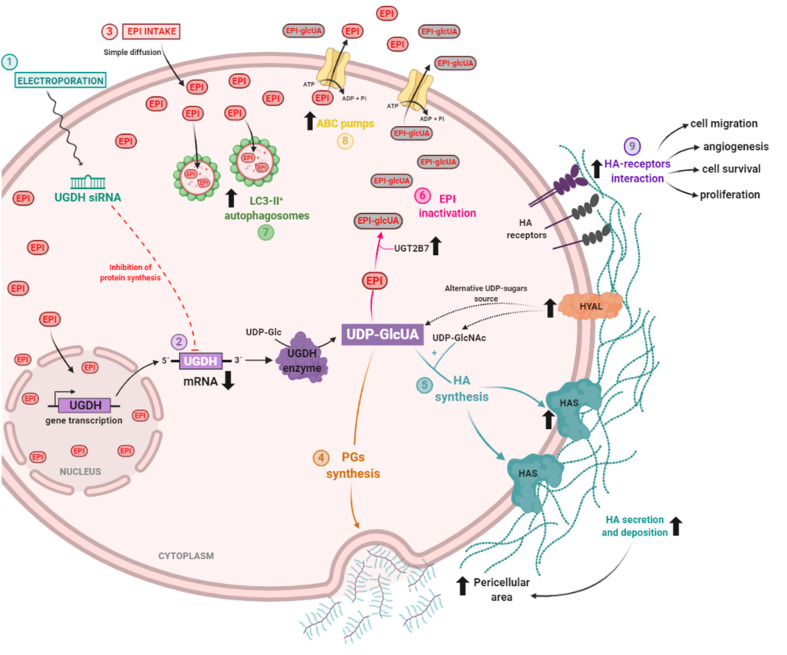
Proposed mechanism of epirubicin resistance in breast cancer cells during UGDH knockdown.

In the present work, we studied the modulation of UGDH using the breast adenocarcinoma cell line MDA-MB-231 as tumor model, with characteristics of lack of response to hormonal therapy and increased aggressiveness (Figure 9). First, we analyzed the frequency of mRNA UGDH expression in different types of solid tumors generated by The Cancer Genome Atlas (TCGA) with the aim to evaluate the relevance of this molecule in solid tumors and validate the use of our model. Besides, our purpose was to postulate the UGDH enzyme and HA-associated genes as prognostic biomarkers in this type of cancer. For that reason, we investigated in published databases the prognostic value related with the expression of main genes involved in HA metabolism (UGDH, HA synthases and hyaluronidases), angiogenesis (VEGF, FGF2, EGF) and drug resistance (ABC drug transporters) in patients with breast cancer stratified by hormone receptor status. We have observed that higher levels of UGDH expression were correlated with a worse prognosis (less survival) in patients with triple-negative breast cancer who have received chemotherapy. Although these results could be controversial comparing to our results, survival analysis performed in silico on triple negative breast cancer patients was carried out with a group of patients who had received chemotherapy, without being able to specify or distinguish between the drugs used for the treatment. The chemotherapy strategy followed in each patient of in silico analysis was not necessarily carried out with anthracyclines or EPI, which are specifically eliminated by glucuronidation and require UGDH. For this reason, it is possible to find discrepancies between the in silico results and those obtained in our study. Similar results were observed in prostate cancer, where downregulation of UGDH promotes androgen-independent tumor cell growth by increasing available levels of intracellular androgen [51], which is why it could be considered as a detection marker for this type of cancer [27]. When we analyzed the correlation between the expression of genes related with UGDH and patient′s survival, we found that increases in the expression of HAS2, HYAL1-2, VEGF and ABCC2 were related to worse prognosis. These results denote the importance of the processes studied in the present work during tumor progression and treatment.

We extended the study by evaluating patients with breast cancer, using samples obtained from patients in the Hospital of our region. In the present study, we analyzed the expression of UGDH mRNA in tumoral (TT) and normal adjacent tissue (NAT) samples obtained from four breast cancer patients previously characterized according to ER, PR, HER2 and Ki67 status in our laboratory [34]. In three patients who were defined as HER2−, we observed an increase in UGDH expression in TT compared to NAT. This result is in concordance with the increase in expression levels of HA, HAS2 and BRCA1 and with previously published data. Although it will be necessary to expand the cohort of studied patients to confirm the results, this analysis complements our in vitro assays, and proposes a starting point for expanding the study to more patients with breast cancer and even extend it to other types of cancer.

Similar results were found when evaluating the expression of UGDH in breast cancer cell lines with different aggressive phenotypes and ER, PR and HER2 status. On the other hand, the patient that showed a decrease in UGDH levels showed also a decrease in HA expression and HAS2 levels [34]. Although further studies are required to understand the function of this enzyme in breast cancer patients and its relation with HA metabolism, these results confirm the data observed in vitro and in silico, and indicate that the mechanisms in which UGDH is involved could be altered during breast cancer progression and treatment.

MDA-MB-231 cells were transfected to introduce the siRNA against UGDH mRNA (1). UGDH translation and synthesis was blocked due to the specific binding of UGDH siRNA to mRNA. UGDH enzyme is responsible for the transformation of UDP-glucose (UDP-Glc) into UDP-glucuronic acid (UDP-GlcUA) (2). Twenty-four hours after transfection, tumor cells were treated with epirubicin (EPI). EPI goes across the cell membrane thanks to its hydrophobic structure. An increase in the intracellular accumulation of EPI was observed, being able to be found both in the cytoplasm and in the nucleus (3).UDP-GlcUA is a precursor for different cellular processes involved in the extracellular matrix and EPI resistance. Specifically, it can be a constituent of different proteoglycans (PGs) and glycosaminoglycans (GAGs), which contributed to the increase of pericellular area of tumor cells (4). In combination with UDP-GlcNAc, by action of hyaluronan syntheses (HASes), UDP-GlcUA is a precursor of the glycosaminoglycan hyaluronan (HA). Unexpectedly, HAS expression and HA synthesis increased after EPI treatment and UGDH knockdown, which also contributed to the increase in the pericellular area (5). Another interesting mechanism we observed was the increase in the expression of HA-degrading enzymes (HYALs). They have been proposed as a new source of UDP-sugars to compensate for the decrease in UGDH enzyme, and therefore UDP-GlcUA availability. Third mechanism in which UDP-GlcUA is involved is the inactivation of EPI. Due to the action of the UGT2B7 transferase, UDP-GlcUA binds to EPI to inactivate its molecule and diminishes EPI activity in tumor cells. During UGDH knockdown, EPI treatment increased UGT2B7 expression favoring EPI inactivation (6). Within the mechanisms activated to avoid EPI activity, an increase in autophagy was detected—a process previously shown to be involved in the development of EPI resistance (7). In agreement, the upregulation of drug efflux pumps (ABC family) was observed in response to EPI treatment (8). Finally, we hypothesized that an increase in the expression and extracellular deposition of HA might affect tumor cells’ behavior and could contribute to development of a resistant phenotype by tumor cells. This may be due to the increase in the interaction between HA and its specific receptors, which might be promoting mechanisms involved in tumor progression, such as angiogenesis, migration, cell survival and proliferation, demonstrated during the present study (9).

Besides, to extend the study to other in vitro models of breast cancer, we investigated the mRNA expression of UGDH from microarray public databases from four breast cancer cell lines with different aggressiveness and hormone receptors status. We determined that the highest expression of this enzyme occurred in MDA-MB-231 cells in basal conditions with respect to other types of triple negative or hormone-sensitive breast cancer cell lines. We consider it important to relate it to the aggressive tumor phenotype that these cells present (triple negative—basal type) in comparison to other cell lines. Furthermore, in response to different concentrations of EPI, breast cancer cells altered the expression of UGDH during resistance acquisition, and with dependence on hormonal receptors expression. This indicates that the enzyme acts as a marker associated with this type of cancer and to determine the response to anthracycline. All the previously performed analyses support our choice to work with these cells as a model for the study of triple negative breast cancer.

Then, we evaluated the role of UGDH during chemotherapy treatment with EPI using the MDA-MB-231 cell line. We have observed that MDA-MB-231 cells express the UGDH enzyme, and we have found a similar effect to the previously reported [2], where the expression was positively regulated in response to EPI treatment. The upregulation of the expression of UGDH could promote the elimination of this cytotoxic drug from tumor cells. It could be related to an increased demand of UDP-GlcUA, which is crucial to conjugate EPI and promote its elimination from tumor cells. Besides, in lung cancer, it has been proposed that an increase in the expression or availability of this enzyme might favor metastasis. This process occurs through the specific interaction between UGDH and HuR protein, which attenuates the UDP-Glc-mediated inhibition of the association of HuR with SNAI1 mRNA, stabilizing it. Increased production of SNAIL initiates the epithelial-mesenchymal transition, thus promoting the migration of tumor cells and metastasis [50].

The key point in our study was to investigate the effect of silencing the UGDH enzyme on the anti-tumoral activity of EPI, while studying pro-tumoral processes such as apoptosis, proliferation, migration, autophagy and angiogenesis. On the other hand, we studied the association of silencing UGDH with the generation of an ECM that favors tumor development and resistance. We observed that silencing of UGDH enzyme combined with EPI treatment did not modify cell viability or cytotoxicity, which means that a possible modulation in the behavior of MDA-MB-231 cells might be a consequence of the effect of reducing the expression of UGDH. We only found significant differences in apoptosis induction after the silencing of UGDH, which seems to diminish the functional ability of EPI as a cytotoxic drug. Contrary to our expectations, we found a significant decrease in the induction of apoptosis in MDA-MB-231 breast cancer cells that had been transfected with siUGDH and after that treated with EPI.

Although MDA-MB-231 cells transfected with siUGDH accumulated a higher amount of EPI, it was not enough to increase the levels of apoptosis observed in the same conditions, moreover apoptosis significantly decreased. These results could be associated with the fact that after silencing UGDH, there is less enzyme available to produce UDP-GlcUA, and UDP-GlcUA will be found in a reduced proportion inside tumor cells. For that reason, there would be less EPI glucuronidation than what is necessary to eliminate the drug. One possible explanation for this result is that intracellular accumulation of EPI does not reflect the activity of this drug or its intracellular localization. Thus, we can hypothesize that despite unconjugated EPI, it could be out of the nucleus avoiding its mechanism of action over nucleic acid and therefore its antitumoral effects. Another alternative hypothesis could be that EPI is in its inactive form, conjugated to UDP-GlcUA from a source that does not depend on UGDH, for example, from the degradation of HA present in the tumor ECM. At this point, we could not detect it because the inactive forms of anthracyclines are also capable of emitting the same fluorescence intensity as the free ones. Another possible explanation for these unexpected results could be the use of a one-sequence siRNA for the inhibition of the expression of UGDH. At this point, we cannot completely rule out the potential off-target effects of siRNA outside of the siRNA target. To further support the results, further experiments using more than one siRNA, a chemical antagonist of UGDH should be performed to confirm our hypothesis.

It is important to highlight that we found by cytometric analysis two well-differentiated populations of tumor cells with different ability to accumulate EPI after silencing the UGDH enzyme. This result could indicate that the population with the least fluorescence intensity represents a population with minor accumulation of EPI and contributes to the whole resistant phenotype observed in our experiments. Different studies will be required to study each population, which will be carried out in our laboratory as an extension of the present study.

We continued evaluating the expression of drug efflux pumps and the specific transferase involved in EPI inactivation, as correlators of a possible mechanism of drug resistance [53,54,55]. As was expected, EPI treatment upregulated the expression of drug efflux pumps. However, the silencing of UGDH plus EPI treatment did not induce a significant modulation of their expression, which could explain the increase in EPI accumulation after siUGDH transfection. In turn, a significant increase in the expression of UGT2B7 was observed in response to UGDH knockdown and EPI treatment. These results indicate that, although EPI efflux was not completely activated, tumor cells upregulated the expression of this enzyme in order to improve the elimination of EPI and avoid the anti-tumoral effect of this drug. As mentioned above, UGT2B7 could use an intracellular UDP-GlcUA from a source not determined yet. All these data, in correlation with a decrease in apoptosis levels despite EPI accumulation, allow us to hypothesize that MDA-MB-231 cells have succeeded in avoiding the potential effect of higher accumulating EPI, favoring mechanisms to develop drug resistance and tumor progress during the anti-tumoral treatment with EPI. In fact, several previous studies have suggested that the appearance of resistance to EPI can occur through different mechanisms, including upregulation of P-glycoprotein, changes in the activity of topoisomerase II and inhibition of apoptotic pathways, among others [56,57,58].

Within mechanisms involved in the development of drug resistance are the modulation of cell survival, proliferation and migration [29,56], and tumor angiogenesis [42]. We did not observe any difference in the activation of Wnt/β-catenin and PI3K/Akt pathways after the knockdown of UGDH and addition of EPI. On the contrary, a significant pro-tumoral effect was visualized in response to the silencing of UGDH when we analyzed angiogenesis and tumor migration. Considering that no differences were detected in VEGF protein levels in supernatants of tumor cells, other factors could be involved in tumor angiogenesis and be related to aggressive phenotypes of different types of cancer cells [44,59]. In this sense, we observed an increase in the expression of VEGF and FGF-2 when cells were transfected with siUGDH and afterward treated with EPI, which are closely involved in the activation of angiogenesis in the tumor environment. In turn, we observed that tumor cells not only conserved their migratory ability under those conditions but also increased migration levels even under UGDH knockdown and EPI treatment. These results are in agreement with the aggressive phenotype of MDA-MB-231 cells and their ability to develop EPI resistance. Moreover, it could be explained by the fact that the migratory capacity is associated with HA turnover, as was observed in changes in the expression of HAS and HYAL enzymes [53]. Together, these results indicate that, despite being under an anti-tumoral treatment and with less availability of the UGDH enzyme, tumor cells activate several mechanisms directly related to tumor progression and drug resistance, despite the cells reducing its glucuronidation and/or elimination.

One of the processes recently observed to be involved with EPI resistance is autophagy. This process is activated under cellular stress and allows the recycling of macromolecules and organelles, inhibiting tumor cell apoptosis [54]. Some evidence indicates that cell autophagy protects MCF-7 breast cancer cells from EPI-induced apoptosis and facilitates the development of EPI resistance [21]. In agreement, in the present study, a positive modulation of autophagy and a decrease of apoptosis in response to EPI treatment have been demonstrated. The effects were also observed in response to UGDH silencing and subsequent EPI treatment. Even more, we observed that increases in the expression of autophagy markers ATG16L1 and LC3-B were associated with poor relapse-free survival in triple negative breast cancer patients. This result would indicate that autophagy is a key process involved in drug response, tumor progression and survival of breast cancer patients. We confirmed these results by performing in vitro analyses, where breast cancer cells favored tumor survival and adaptation to the stress generated by the anti-tumoral treatment with EPI activating autophagic processes. Moreover, we observed that the lack of UGDH could support the development of resistance to EPI through the process of autophagy.

Furthermore, we consider it important to highlight that tumor cells are able to modulate their extracellular microenvironments to avoid drug action [55]. According to the role of UGDH in HA expression, there are controversial data regarding the effect on the modulation of its expression. First, it has been demonstrated that a diminished function of the UGDH enzyme (either by siRNA or 4-MU), in aortic smooth muscle cells [4,6] and human keratinocytes [60], significantly reduces the production of HA. According to previous results, Wang et al. demonstrated that inhibition of UGDH expression significantly decreased the invasive capacity of HCT-8 colorectal carcinoma cells in combination with a reduction in the expression of different GAGs [2]. However, the experiments were carried out without a chemotherapeutic agent, such as an anthracycline.

We continued analyzing the effect of UGDH knockdown plus EPI on the metabolism of HA. Although we observed that MDA-MB-231 cells had less availability of UGDH to synthesize UDP-GlcUA, the cells were able to favor the expression of ECM components mainly composed of HA, as was observed in the particle exclusion assay. However, we found no differences in the concentration of HA present in the extracellular medium. We continue analyzing the expression of HASes and HYALs enzymes as essential components of HA metabolism. The silencing of UGDH combined with EPI treatment increased the expression of HAS2 and HAS3. These results were in line with the data obtained in the particle exclusion assay and the HA ELISA like-assay, considering that HAS3 is responsible for synthesizing the HA that is generally retained in the plasma membrane. At the same time, HAS2 is mostly involved in the synthesis of HA released into the cellular medium [25,26].

Conversely, we observed a lesser increase in HAS2 expression in accordance with a slight modulation of soluble HA levels. Besides, it would be interesting to study the molecular weight of HA produced by tumor cells, since it can have differentiated functions [26]. Furthermore, the unexpected increase in HYALs expression considering higher pericellular area could be explained taking into account that, in the absence of UGDH, breast cancer cells require a new source of UDP-GlcUA to synthesize HA (and other GAGs and PGs). Consequently, MDA-MB-231 cells could activate the expression of the enzymes that degrade HA to favor this process, since it can digest its precursors leaving them available for other processes as EPI glucuronidation. In agreement with our study, it has been recently demonstrated that the depletion of UDP-GlcUA inhibits mesenchymal-like properties, including cellular invasion and colony formation in vitro, and tumor growth and metastasis in vivo [60]. In fact, we previously demonstrated that the addition of low molecular weight HA during treatment with the anthracycline DOX favors the tumor through increased migration of endothelial cells [29].

Our results are in line with previous studies in prostate cancer, where it was shown that when overexpressing the UGDH enzyme during androgen treatment (similarly eliminated by glucuronidation), the synthesis of HA was not stimulated, although HAS3 expression was increased [51]. However, it has been determined that sugars attached to UDP (UDP-sugars) influence the carrying of HAS3 to the plasma membrane of melanoma cells, thereby affecting the function of that enzyme and finally, HA synthesis [61,62,63,64]. In fact, the synthesis of this GAG can be regulated by cell metabolism because glucose levels have a substantial impact on the concentration of UDP-sugars. Therefore, it would be important to determine the activity of HYALs enzymes, and the analysis of UDP-sugars available in MDA-MB-231 cells in order to determine whether the increase in the expression implicates an increase in the activity of enzymes.

Although the expression of UGDH at the protein level was not analyzed during the present work, an approximation was carried out through bioinformatics tools. For that reason, in order to evaluate whether the results obtained on a genetic level correlate with protein functions and cell effectors, STRING analysis was performed. It showed that PI3K/AKT and Wnt signaling pathways connect the mechanisms of autophagy, angiogenesis, drug resistance and HA metabolism evaluated in the present work. UGDH is directly involved in HA synthesis and glucuronidation reactions. Indeed, UGT transferase UGT2B7 interacts with the studied drug efflux pumps, showing a relation between the responses to chemotherapy that requires glucuronidation and drug efflux. As expected, the enzymes related to HA metabolism (HASes and HYALs) were interrelated with each other, showing a strict regulation. The rest of the molecules are also interconnected. We know that further research is needed to confirm these novel findings, but this should support new studies of UGDH in breast cancer and other types of tumors.

In summary, we suggest that a specific tumor microenvironment and ECM benefit the intracellular accumulation of EPI. However, this event would not necessarily increase the activity of the drug and the consequent efficiency of the chemotherapy treatment. Tumor cells demonstrated to be able to respond to EPI treatment by activating crucial cellular processes, such as autophagy, angiogenesis and cell migration, and by leading to the re-organization of ECM components such as HA, which favors tumor progression. In this process, the role of UGDH is crucial, making it possible to be proposed as a marker of tumor progression during chemotherapy in breast cancer patients.

## Data Availability

The authors declare that all data supporting the findings of this study are available within the article. Besides, the datasets generated during and/or analyzed during the current study are available from the corresponding author on reasonable request (laualaniz@comunidad.unnoba.edu.ar).

## Supplementary Materials

The following are available online at https://www.mdpi.com/2218-273X/11/2/246/s1, Figure S1: Modulation of autophagy detected by confocal microscopy.
