# Supplementary material for: Initial Identification of UDP-Glucose Dehydrogenase as a Prognostic Marker in Breast Cancer Patients, Which Facilitates Epirubicin Resistance and Regulates Hyaluronan Synthesis in MDA-MB-231 Cells
Source: Biomolecules. 2021 Feb 9;11(2):246. doi: 10.3390/biom11020246 (PMC7914570; doi:10.3390/biom11020246)
Supplement: Supplementary file 1 [file biomolecules-11-00246-s001.pdf]

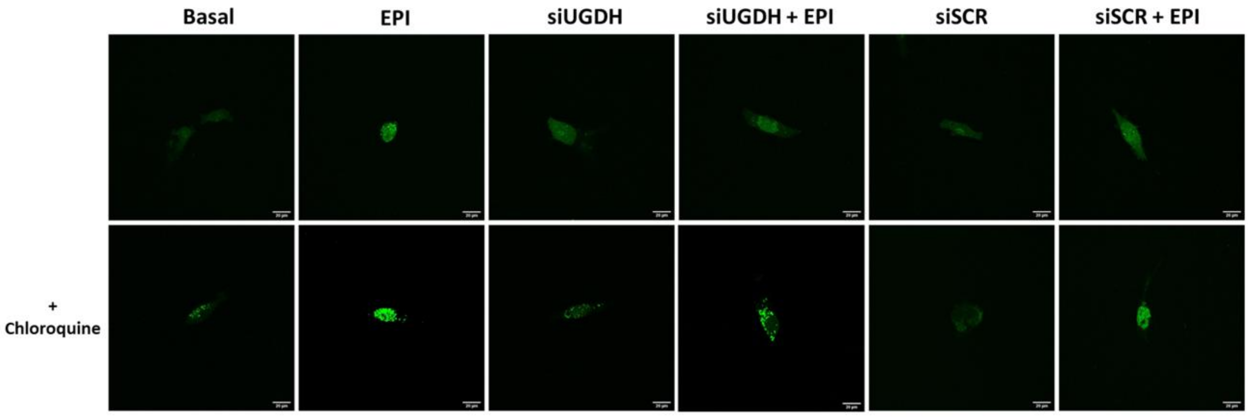

Supplementary figure 1. Modulation of autophagy detected by confocal microscopy. MDA-MB-231 cells were co-transfected with a specific siRNA against UGDH gene (siUGDH) or a random sequence siRNA as negative control (siSCR) and 2 μg of a specific LC3-GFP construct. After 24h, 1 μM EPI (EPI) was added to complete 48h of incubation. To analyze the formation of autophagosomes, the fluorescence emitted by GFP was evaluated through confocal microscopy adding chloroquine as a specific inhibitor of autophagy.
